# Computational approaches to address data challenges in intellectual and developmental disabilities research

**DOI:** 10.1186/s11689-022-09472-8

**Published:** 2023-01-12

**Authors:** Daifeng Wang, John R. Pruett

**Affiliations:** 1 Waisman Center, University of Wisconsin-Madison, Madison, WI 53705 USA; 2 Department of Biostatistics and Medical Informatics, University of Wisconsin-Madison, Madison, WI 53076 USA; 3 Department of Computer Sciences, University of Wisconsin-Madison, Madison, WI 53076 USA; 4 Department of Psychiatry, Washington University School of Medicine in St. Louis, Saint Louis, MO USA; 5 Department of Radiology, Mallinckrodt Institute of Radiology, Washington University School of Medicine in St. Louis, Saint Louis, MO USA; 6 Department of Psychological & Brain Sciences, Washington University in St. Louis, Saint Louis, MO USA

Advanced technologies such as gene sequencing and brain imaging have generated a variety of complex datasets for research on neurodevelopmental disorders. These datasets can be valuable resources to help understand biological mechanisms, monitor disease progression, and improve clinical diagnosis in neurodevelopmental disorders. Though computational and statistical approaches have been successfully applied in other fields, the computational analysis of large neurodevelopmental disorder datasets remains exceedingly challenging. To this end, the fifth IDDRC special thematic series of the *Journal of Neurodevelopmental Disorders* collects peer-reviewed research and review articles focusing on “Computational Biology and Neurodevelopmental Disorders,” from the NICHD-funded Eunice Kennedy Shriver Intellectual and Developmental Disabilities Research Centers (IDDRCs) across the nation. The articles in this issue introduce recent computational and statistical studies in IDDs; present emerging data types, such as next-generation sequencing data; and highlight the great potential of advanced computational methods such as machine learning to facilitate IDD research. Below is a brief summary of what is featured in each article.

Increasing next-generation sequencing data across multi-omics necessitate computational analyses for a deeper understanding of cellular and molecular mechanisms at the genome-wide level. For instance, Veatch and colleagues analyzed whole exome sequence data from >2000 individuals and calculated genetic risk for dysfunction in pleiotropic biological processes (10.1186/s11689-022-09448-8). They predicted protein-damaging variants (PDVs) in genes currently implicated in either autism spectrum disorder (ASD) or sleep dysfunction in typically developing children, concluding that genetic dysfunction possibly impacting the development of the cerebral cortex may affect sleep by disrupting sleep homeostasis. In addition to protein-coding genes, Stein and Won discussed the topic of massively parallel reporter assays (MPRA) to experimentally validate genetic variants associated with neurodevelopmental disorders (10.1186/s11689-022-09461-x). MPRA is an emerging method to functionally validate thousands of non-coding regulatory elements simultaneously using high-throughput sequencing and barcode technology and thus can help narrow down potential underlying genetic causes of neurodevelopmental disorders by screening thousands of sequences in one experiment. Stein and Won also describe future directions involving this technique, such as applications of MPRA for studies of gene-by-environment interactions and pharmacogenetics.

At the RNA level, Zheng and colleagues analyzed recent single-cell RNA-seq data (>104k cells) to characterize cell-cell communications in ASD and controls (10.1186/s11689-022-09441-1). They found that the cell communication differences in ASD not only largely involve neurons, as both signal senders and receivers, but glia also contribute to the communication disruption. In addition, excitatory and inhibitory neurons are involved in multiple intercellular signaling processes that exhibit increased strengths in ASD, such as NRXN and CNTN signaling. Genes in the affected signaling pathways are enriched for axon guidance, synapse organization, neuron migration, and other critical cellular functions. Furthermore, those genes are highly connected to and enriched for genes previously associated with ASD risk. To integrate disease variants with gene expression data, Fry, Li, Santos, and colleagues integrated latent profile analysis with GWAS and eQTL data to characterize cognitive function at age 10 in children born extremely preterm and shed light on genetic mechanisms underlying cognitive impairment (10.1186/s11689-022-09429-x). They identified two loci reaching genome-wide significance, as well as potential functional genes that could impact cognitive impairment for extremely preterm-born children. Finally, at the protein level, Kratimenos and colleagues used both experimental and computational methods to identify molecular events critical for the onset of excitotoxicity-induced apoptosis in the cerebral cortex of newborn piglets (10.1186/s11689-022-09431-3). This study captured important molecular trends caused by hypoxia in the piglet brain. Incorporating the action of Src kinase inhibitor PP2 further validated the discovery and enabled predictive analysis of the effect of hypoxia on CaMKK2, providing a feasible framework for drug efficacy studies in translational models of neonatal brain injury for the prevention of IDDs.

In addition to the sequencing data, computational approaches can be applied to other unmet classical data types including those from neuroimaging and neurophysiology. For instance, Lee, Cho, and colleagues employed multivariate distance matrix regression (MDMR) on structural MRI volumes to study intelligence quotient (IQ) trajectories in ASD (10.1186/s11689-022-09460-y). Their research team had previously identified subgroups with disparate developmental trajectories in IQ. The authors used this MDMR computational approach to determine that the default mode network (DMN) structure predicted changing IQ across development, while the fronto-parietal network (FPN) structure correlated with current high IQ. Cohen contributes a paper about lesion network mapping, an approach that may help identify brain networks linked to complex symptoms in IDDs (10.1186/s11689-022-09433-1). This approach combines computational analysis with neuromodulation, including the use of real-time MRI-based neurofeedback and transcranial magnetic stimulation (TMS). Existing datasets may be leveraged to generate hypotheses for imaging studies in IDDs, where these neuromodulation approaches may be applied. There is obvious and great potential for this approach—above more basic correlative scientific approaches—to help guide future randomized clinical trials, with the goal of improving prospects for successful translation. Arnett and Flaherty took an advanced statistical approach to provide a framework and guide-chart for future studies involving mixture modeling approaches that are suitable for typical sample sizes that are found in research on IDDs (10.1186/s11689-022-09454-w). Their methods draw from structural equation modeling to augment latent profile analysis, as informed by criteria that are weighted by the interpretation of existing scientific evidence. They bring this approach to the study of 120 children with attention-deficit/hyperactivity disorder (ADHD) who have been characterized with neuropsychological measures and EEG metrics. This approach allowed them to arrive at a five-class stable solution and to attempt extrapolation from a model based on behavioral data to identify distinct patterns in resting EEG power profiles.

Complex phenotypic data have also been studied in IDD research. Zhou, Wang, and colleagues used machine learning techniques such as natural language processing and clustering analysis to analyze clinical notes in the electronic health record (EHR) from >24,000 individuals with ASD and non-ASD psychiatric disorders (10.1186/s11689-022-09442-0). They identified the largest ASD EHR terminology set to date, with 3336 ASD terms linking to 1943 unique medical concepts. Furthermore, these terms could be used in a diagnostic pipeline to differentiate individuals with ASD from individuals with other psychiatric disorders. Quinde-Zlibut and colleagues contributed a paper about the use of automated digital video facial coding analysis and unsupervised clustering to identify potential ASD subsets (10.1186/s11689-022-09451-z). The authors use this digital phenotyping approach to identify a subset of ASD individuals with high spontaneous facial expressions regardless of the valence of emotional images that they observe. They failed to find evidence for incongruous and inappropriate facial expressions in ASD, but they identified a negative trend for expressiveness and emotion recognition.

Finally, Gupta and colleagues review the current state of machine learning techniques as they are utilized in the field to uncover potential mechanistic contributions to IDDs (10.1186/s11689-022-09438-w). This thoughtful review considers the power afforded by leveraging information from multiple modalities, including neuroimaging data, phenotypic data (digital, speech audio), electronic health records, and multi-omics data. Existing publicly available data repositories provide tremendous opportunities for the application of these analytics. This review additionally covers concepts, gaps to be filled, and indexes available machine learning implementations—mentioning the need to evaluate the performance of trained machines on completely unseen data. Gupta and colleagues highlight the particular power of machine learning for predictive outcome classification, using ASD and cerebral palsy (CP) as example conditions. The authors also make a plea for more investigation in fields less well represented in the IDD literature (CP, fragile X, and Down syndrome), which features a large number of reports about ASD.

These papers exemplify leading innovations in the application of computational neuroscience and advanced analytic approaches to address fundamental issues in IDD basic and translational research. One major crosscutting theme is the goal of using computational neuroscience to aid future, personalized medicine approaches for IDDs. Digital phenotyping methods are applicable for the study of subjects and patients with IDDs at all ages. In addition, specific examples highlighted in these papers are test-use-cases for future extension to other disorders and for employment with large, multimodal data sets that are increasingly being assembled through consortia and public data sharing resources. For example, Gupta and colleagues highlight how machine learning may help us to identify previously unknown disease patterns from electronic health records, generate mechanistic hypotheses to be tested in intervention trials, and identify patterns in biological networks that may facilitate efforts in drug repurposing and to improve health in IDDs. The breadth and depth of approaches in these papers highlight an important aspect of research being conducted by our national IDDRC network. This 2022 *Journal of Neurodevelopmental Disorders* Special Section on Computational Neuroscience of IDD sets the stage for a future follow-up issue that will, no doubt, showcase further advances in computational neuroscientific approaches, analytics, multimodal data processing, and—with hope—collaboration across the IDDRCs on these efforts.

## Data Availability

Not applicable.

